# Identification of pyroptosis-related genes and potential drugs in diabetic nephropathy

**DOI:** 10.1186/s12967-023-04350-w

**Published:** 2023-07-21

**Authors:** Meng Yan, Wenwen Li, Rui Wei, Shuwen Li, Yan Liu, Yuqian Huang, Yunye Zhang, Zihao Lu, Qian Lu

**Affiliations:** 1 Jiangsu Key Laboratory of New Drug Research and Clinical Pharmacy, Xuzhou Medical University, Xuzhou, China; 2 Department of Clinical Pharmacology, School of Pharmacy, Xuzhou Medical University, No. 209 Tongshan Road, Xuzhou, 221004 Jiangsu China; 3 Jiangsu Key Laboratory of Brain Disease and Bioinformation, Research Center for Biochemistry and Molecular Biology, Xuzhou Medical University, Xuzhou, China; 4 Key Laboratory of Genetic Foundation and Clinical Application, Department of Genetics, Xuzhou Engineering Research Center of Medical Genetics and Transformation, Xuzhou Medical University, Xuzhou, China

**Keywords:** Diabetic nephropathy, Pyroptosis, Lasso regression, Enrichment analysis, Drug–gene prediction

## Abstract

**Background:**

Diabetic nephropathy (DN) is one of the serious microvascular complications of diabetes mellitus (DM). A growing body of research has demonstrated that the inflammatory state plays a critical role in the incidence and development of DN. Pyroptosis is a new way of programmed cell death, which has the particularity of natural immune inflammation. The inhibition of inflammatory cytokine expression and regulation of pathways related to pyroptosis may be a novel strategy for DN treatment. The aim of this study is to identify pyroptosis-related genes and potential drugs for DN.

**Methods:**

DN differentially expressed pyroptosis-related genes were identified via bioinformatic analysis Gene Expression Omnibus (GEO) dataset GSE96804. Dataset GSE30528 and GSE142025 were downloaded to verify pyroptosis-related differentially expressed genes (DEGs). Least absolute shrinkage and selection operator (LASSO) regression analysis was used to construct a pyroptosis-related gene predictive model. A consensus clustering analysis was performed to identify pyroptosis-related DN subtypes. Subsequently, Gene Set Variation Analysis (GSVA), Gene Ontology (GO) function enrichment analysis and Kyoto Encyclopedia of Genes and Genomes (KEGG) pathway analysis were conducted to explore the differences between DN clusters. A protein–protein interaction (PPI) network was used to select hub genes and DGIdb database was utilized to screen potential therapeutic drugs/compounds targeting hub genes.

**Results:**

A total of 24 differentially expressed pyroptosis-related genes were identified in DN. A 16 gene predictive model was conducted via LASSO regression analysis. According to the expression level of these 16 genes, DN cases were divided into two subtypes, and the subtypes are mainly associated with inflammation, activation of immune response and cell metabolism. In addition, we identified 10 hub genes among these subtypes, and predicted 65 potential DN therapeutics that target key genes.

**Conclusion:**

We identified two pyroptosis-related DN clusters and 65 potential therapeutical agents/compounds for DN, which might shed a light on the treatment of DN.

**Supplementary Information:**

The online version contains supplementary material available at 10.1186/s12967-023-04350-w.

## Introduction

Diabetic nephropathy (DN), also known as diabetic kidney disease (DKD), is one of the most serious chronic microvascular complications of diabetes mellitus (DM) [1]. It is reported that up to 50% of patients will have kidney disease in the process of DM [2]. The early clinical manifestations of DN are characterized by glomerular hyperfiltration rate and clinical microalbuminuria [3]. As the disease progresses, patients will have a continuous large amount of albuminuria, with glomerular nodular lesions in renal pathology, eventually developed into end stage renal disease (ESRD). DN is the leading cause of ESRD, which accounts for a large proportion of patients undergo kidney transplant treatment in many populations [2]. From 1990 to 2019, incident DN cases had risen by 156.5% and deaths by 172.4%, which means DN has become one of the public problems that severely threaten the health of people [4].

The pathogenesis of DN, which is extremely complicated and multifactorial. Oxidative stress, angiotensin II (Ang-II) and inflammation are recently considered to play an important role [5]. Oxidative stress induced by hyperglycemia can cause direct damage to glomerular cells, leading to albuminuria and tubular interstitial fibrosis [6]. Intrarenal Ang II can cause renal injury by increasing glomerular capillary permeability and promoting mesangial cell proliferation [7, 8]. Recent evidence suggests that persistent kidney inflammation plays an important role in the development and progression of DN [9, 10]. Inflammatory cells, synthesize and secret proinflammatory cytokines in the kidney, can directly damage the renal cell and trigger the epithelial to mesenchymal transition (EMT). More importantly, there are overlaps between those pathways and mechanisms and this is the reason why the exact pathogenesis of DN is still not fully understood.

Pyroptosis, recently, has been identified as a novel form of programmed cell death accompanied by the release of numerous pro-inflammatory factors such as IL-1β and IL-18 [11]. The signal transduction mechanisms of cell pyroptosis include caspase-1 dependent classical pathway, caspase-4/5/11 dependent non-classical pathway and caspase-3 dependent pathway [12]. All patterns are characterized by the formation of pores on the cell membrane and the release of cell contents mediated by gasdermin (GSDM), and eventually lead to cell swelling and rupture, thereby triggering a cascade amplification of the inflammatory response [13, 14]. Increasing studies have demonstrated that pyroptosis is closely linked to renal injury in the development of DN [15–17], and many compounds have been reported could alleviate the development of DN through inhibiting pyroptosis [18–20]. However, the research into cell pyroptosis in DN is still in its infancy. Moreover, pyroposis associated molecular subtypes have been found in many diseases [21, 22]. Patients exhibit diverse clinical characteristics in different subtypes such as diagnosis, prognosis, etc. Therefore, the purpose of our study is to identify pivotal pyroptosis related targets and subtypes of DN, and then to predict potential therapeutic drugs. Further investigations about pyroptosis may contribute to the discovery of a new therapeutic strategy for DN.

**Fig. 1:**
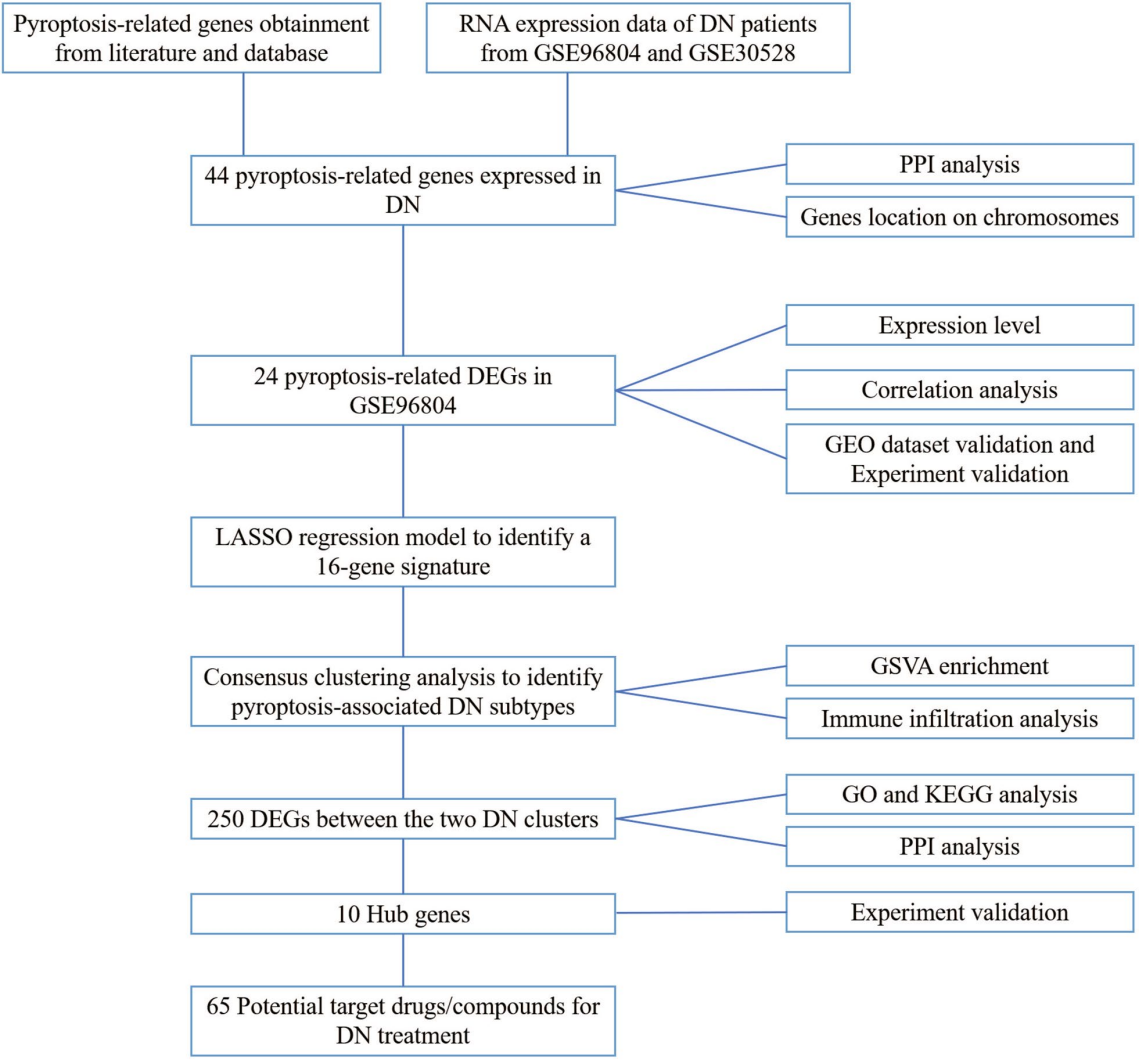
Schema of the study

In the current study, we performed bioinformatics analysis to determine the expression profiles of pyroptosis-related genes between normal kidney and DN tissues, and identified two types of DN subgroups according to pyroptosis-related genes. And we found that these two patterns have different immune infiltration characteristics, and the differentially expressed genes (DEGs) between subtypes are enriched in inflammation related signaling pathways. Moreover, hub genes of DEGs between clusters were selected by the Cytoscape software and validated using qRT-PCR in glomerular mesangial cells. Candidate drugs for DN treatment were screened in the Drug Gene Interaction Database (DGIdb) on the basis of the hub genes identified above. The detailed schematic of the workflow in the current study is shown in Fig. 1. In summary, our data may provide additional evidence for the diagnosis and treatment of DN.

## Materials and methods

### Obtainment and preprocessing of DN datasets

Gene expression data of DN patients were obtained from the GEO database (http://www.ncbi.nlm.nih.gov/geo). Three datasets (GSE96804, GSE30528and GSE142025, transcriptome analysis of renal tissue in patients with DN, and they were used in many studies about DN) were chose for our next analysis. The detail information of selected datasets is shown in Table 1. Kidney tissue samples from DN patients in the first two datasets were microdissected glomeruli from kidney, and the corresponding samples in the last dataset were whole kidney tissue. All control human kidney samples were obtained from the unaffected portion of tumor nephrectomies.

**Table 1:** GSE datasets referenced in this study

GSE dataset	Organism	Sample number	PMID	Platform
GSE96804	*Homo sapiens*	Control:20 DN: 41	30511699 29242313	GPL17586-[HTA-2_0] Affymetrix Human Transcriptome Array 2.0 [transcript (gene) version]
GSE30528	*Homo sapiens*	Control: 26 DN: 9	21752957 26190114	GPL571-[HG-U133A_2] Affymetrix Human Genome U133A 2.0 Array
GSE142025	*Homo sapiens*	Control: 9 Advanced DN: 21	31578193 32086290	GPL20301-Illumina HiSeq 4000 (Homo sapiens)

### Identification of differentially expressed pyroptosis-related genes

We extracted pyroptosis-related genes from literature 33828074 [23] and the Molecular Signatures Database (MSigDB) database, and they are presented in Additional file 1: Table S1 and Fig. S1. Pyroptosis-related genes that were differentially expressed in GSE96804 dataset were identified using the “limma” R package with *P* < 0.05 and |logFC| > 1.

### Correlation analysis

The expression correlation of 24 pyroptosis-related DEGs in all samples and DN samples was calculated. The correlations were performed by Pearson's correlation analysis using rcorr function in the “hmisc” R package and visualized with using the “corrplot” R package.

### LASSO regression

Least absolute shrinkage and selection operator (LASSO) regression (“glmnet” R package) was applied to construct pyroytosis-related genes predictive model of DN. By constructing a penalty function, the coefficients of the relatively unimportant variables can become zero and are eventually excluded from the model. Then, pyroytosis-related genes that had a greater impact on DN were selected and the corresponding regression coefficients were obtained. Here, the binary discrete variable model and the default parameters of R package glmnet are selected to construct the model, with a regression penalty score of 0.01561553. The risk score of the predictive model was calculated after standardization of the GEO expression data, and the risk score formula was as follows: Risk Score= ${\sum }_{i}^{n}Xi\times Yi$ (*X*: coefficients, *Y*: gene expression level).

### Consensus clustering

Consensus clustering, a method to identify molecular subtypes based on approximate number of clusters, was utilized to discover DN subgroups relating to the expression of pyroptosis-related regulators by the k-means method. The maximum number of categories to be evaluated is 10, and the number of iterations for each k is 50. The Euclidean distance is selected as the clustering distance. The number of clusters was calculated by the consensus clustering algorithm using the “ConsensuClusterPlus (1.58.0)” R package. Graphical results included heatmap of the consensus clustering, consensus cumulative distribution function (CDF) plots, and delta area plots.

### Gene set variation analysis (GSVA)

GSVA analysis, one of the GSEA algorithms, could explore the difference in biological pathways between distinct pattern clusters according to the enrichment score. “GSVA (1.16.0)” R Package was applied to perform functional enrichment analysis of DN disease samples in GSE96804 to obtain the enrichment pathways. We downloaded “c2.cp.kegg.v7.4.symbols.gmt” from MsigDB database to carry out the analysis. Adjusted *P* value < 0.05 was considered to suggest statistical difference between distinct clusters.

### Functional enrichment analysis

The biological functions of identified 250 differentially expressed genes among distinct clusters were assessed using the Gene Ontology (GO) function enrichment analysis and Kyoto Encyclopedia of Genes and Genomes (KEGG) pathway analysis with the “clusterProfiler (3.18.1)” R package. For GO analyses, enriched biological processes (BPs), molecular functions (MFs), and cellular components (CCs) were assessed. Adjusted *P* < 0.05 was considered a statistically significant difference in enrichment analysis, and the top 20 of each analysis were extracted for visualization.

### Protein–protein interaction (PPI) network analysis

PPI networks were constructed by importing genes into the Search Tool for the Retrieval of Interacting Genes (STRING, www.string-db.org), with those interactions with a combined score > 0.5 being used for network construction. Cytoscape (version 3.7.2) was used to visualize the network, while the cytoHubba plugin was used to rank genes within this network based on their degree centrality values. The algorithm selected in cytoHubba is Betweenness. Hub genes were considered to be those with the top 10 highest scores.

### Predication of drug–gene interaction

The Drug–Gene Interaction Database (DGIdb, http://www.dgidb.org/) is an online database of drug–gene interaction data aggregated from various sources, including several drug databases (DrugBank, PharmGKB, ChEMBL), clinical trial databases, and literature from PubMed. Information on over 40,000 genes and 10,000 drugs was collected and organized, involving over 100,000 drug-gene interactions. The selected key genes that were considered potential pharmaceutical targets for DN treatment were imported into DGIdb to explore existing drugs or small organic compounds. For each drug-gene interaction, the reliability of the interaction is evaluated based on the evidence supporting the interaction from relevant drug databases such as DrugBank. Based on the interaction score, we selected the top 30 drugs as potential therapeutic drugs for DN. Results were displayed using the “ggplot2 (3.2.1)” and “ggalluvial (0.11.1)” R packages.

### RNA extraction and quantitative real-time polymerase chain reaction (qRT-PCR)

The total RNA from glomerular mesangial cells was extracted by Trizol reagent (Tiangen biotech, Beijing, China), and RNA concentration was measured using NanoDrop One/OneC (Thermo scientific, USA). Subsequently, reverse transcription of RNA into cDNA was executed with HiScript^®^ II Q RT SuperMix for qPCR (Vazyme, Nanjing, China) according to the standard instructions. LightCycler^®^ 96 Instrument (Roche, Switzerland) was performed for qRT-PCR to detect mRNA expression. All the primers were synthesized by Sangon Biotech (Sangon, Shanghai, China). The relative expression level of mRNA was calculated by 2^−∆∆Ct^ method and three replicate experiments were involved. All specific primers were shown in Table 2.

**Table 2:** Primers used for real-time PCR

Gene	Species	Primer sequence (5′ to 3′)
ALB	Mouse	Forward: TGTGTTGCCGATGAGTCTGC
		Reverse: ACGGAGGTTTGGAATGGCAC
C1QB	Mouse	Forward: CTGCCTCTAGGGACCCAGACTTC
		Reverse: ATGGAATCCTGGTGTTCTGTGATGC
CASP1	Mouse	Forward: GAGAAGAGAGTGCTGAATCAG
		Reverse: CAAGACGTGTACGAGTGGTTG
CASP8	Mouse	Forward: GGATGTTGGAGGAAGGCAATCTGTC
		Reverse: GGGAGAAATCTGGGCATTGTCTGG
CD44	Mouse	Forward: CCTGGCACTGGCTCTGATTCTTG
		Reverse: CTGTCTTCCACCGTCCCATTGC
COL3A1	Mouse	Forward: GGTGTAAAGGGTGAACGTGGTAGTC
		Reverse: TTGCCAGGAGGACCAGGAAGAC
EGF	Mouse	Forward: GCCAGGGATGGAAACCTGTG
		Reverse: ATCCCGTCTCCTTCGTAGCC
FN1	Mouse	Forward: GAGCAAGCCTGAGCCTGAAG
		Reverse: GGCGCTCATAAGTGTCACCC
GPR183	Mouse	Forward: TCTGCCTGTCCGTCTGGATTCTG
		Reverse: GTGGTCTTGTCTCCCTCCTCCTTAG
HPGDS	Mouse	Forward: CAATCCACCAGAGCCTCGCAATAG
		Reverse: CATGAAGTCATCCAGCGTGTCCAC
IL18	Mouse	Forward: CAAAGAAAGCCGCCTCAAACCTTC
		Reverse: TTGACGCAAGAGTCTTCTGACATGG
NEGR1	Mouse	Forward: AGCCTTGCGTTGACACTATCTTCTG
		Reverse: AGCCATCAGCACTCCCAGAGAC
SPON1	Mouse	Forward: GGACAGAAGGGCAAGGACAGTAATG
		Reverse: GGGTAAGGCTCGGAGGTCAGTC
TP53	Mouse	Forward: GGACCCTGGCACCTACAATGAAATC
		Reverse: CCCTGGAGGATATGGACCCTATGAG
β-actin	Mouse	Forward: AGAGGGAAATCGTGCGTGAC
		Reverse: CAATAGTGATGACCTGGCCGT

### Statistical analysis

All statistical analyses were carried out in R 4.1.1 and GraphPad Prism 8. Univariate and multivariate logistic regression analyses were utilized to assess the diagnostic value of the predictive mode. All statistical tests were two-sided, and *P* < 0.05 represents significant difference.

## Results

### Defining of the expression of pyroptosis-related genes in DN

**Fig. 2:**
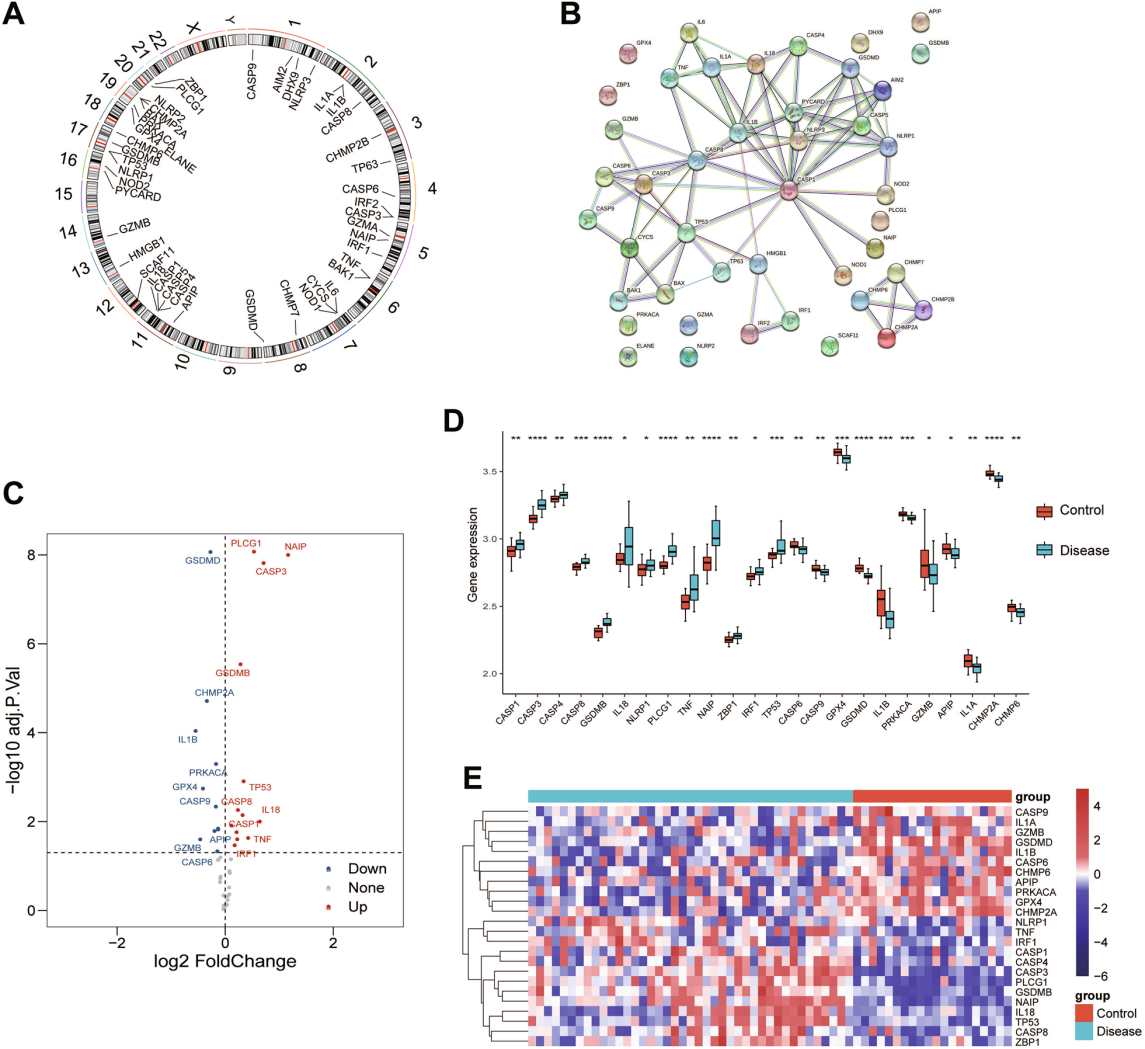
Characteristics and differences of pyroptosis-related genes in DN. **A** The location of 44 pyroptosis-related genes on 23 chromosomes. **B** PPI network showing the interactions of the pyroptosis-related genes (interaction score = 0.9). **C** A volcano plot of the 24 differentially expressed pyroptosis-related genes in the GSE96804 dataset. Upregulated, red; downregulated, blue. **D** A box plot of the above 24 pyroptosis-related genes. Normal, red; Disease, blue. *P* values were shown as: *P < 0.05; **P < 0.01; ***P < 0.001. **E** An expression heatmap corresponding to the 24 pyroptosis-related genes

We obtained 57 pyroptosis-related genes from literature 33,828,074 [23] and Reactome gene sets and GOBP gene sets from MSigDB database (https://www.gsea-msigdb.org/gsea/msigdb/index.jsp) [24, 25]. Then we explored the expression of the 57 pyroptosis-related genes in DN and normal kidney tissues using the GSE96804 and GSE30528 datasets. A total of 44 pyroptosis-related genes were expressed in DN. Figure 2A presents the location of these 44 pyroptosis-related genes on chromosomes. To further explore the interactions of these pyroptosis-related genes, we conducted a PPI analysis using STRING platform, and the results are shown in Fig. 2B. The minimum required interaction score for the PPI analysis was set at 0.900 (the highest confidence). Next, the “limma” R package in Bioconductor was used to identified the differentially expressed pyroptosis-related genes in the GSE96804 with a *P* < 0*:*05 and a |logFC|> 1. A total of 24 differentially expressed pyroptosis-related genes were identified (Fig. 2C–E). Among them, 13 genes (CASP1, CASP3, CASP4, CASP8, GSDMB, IL18, NLRP1, PLCG1, TNF, NAIP, ZBP1, IRF1 and TP53) were upregulated while 11 other genes (CASP6, CASP9, GPX4, GSDMD, IL1B, PRKACA, GZMB, APIP, IL1A, CHMP2A and CHMP6) were downregulated.

**Fig. 3:**
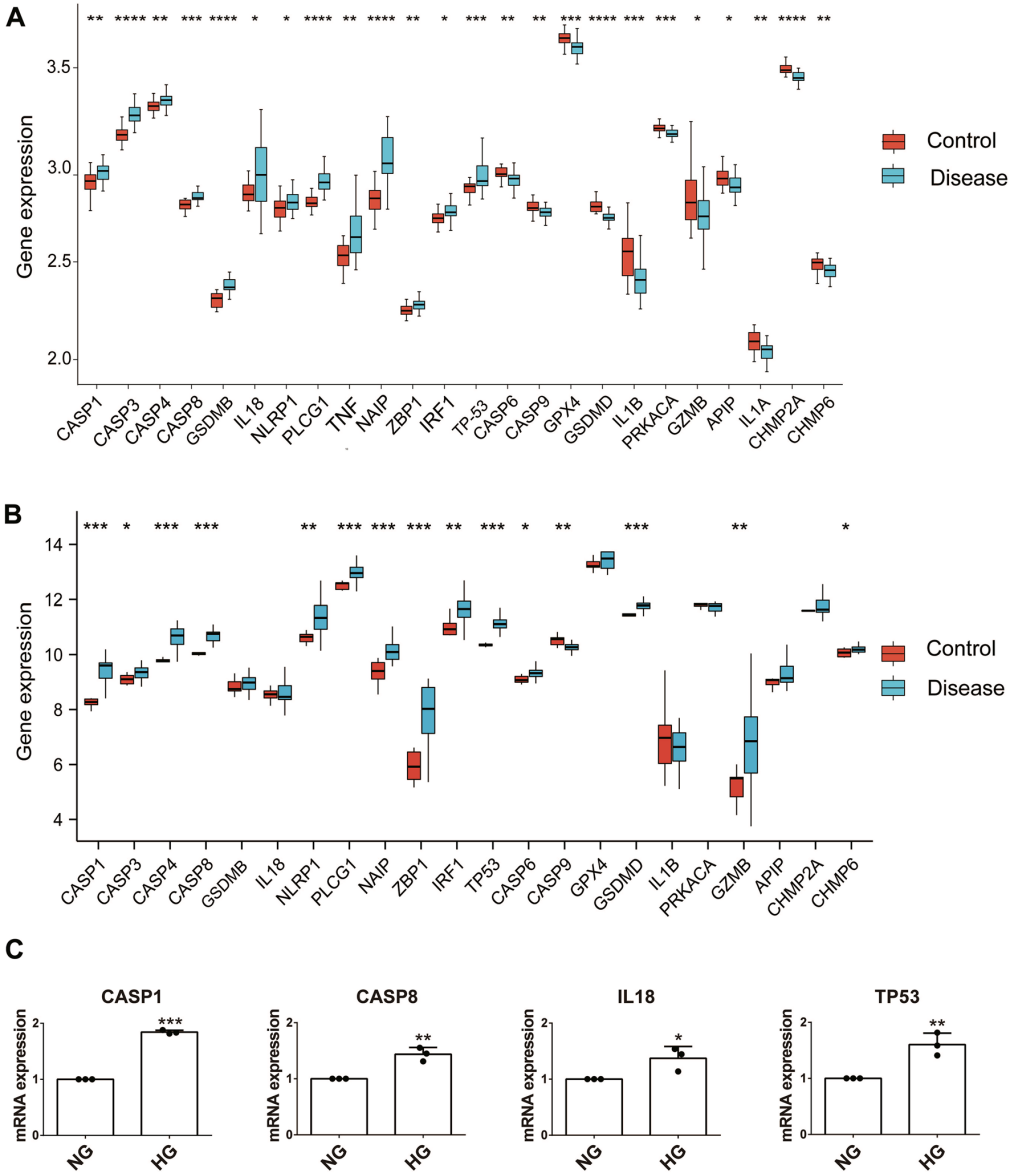
Validation of 24 differentially expressed pyroptosis-related genes from dataset GSE96804. **A** Expression level of 24 pyroptosis-related DEGs in GSE30528. **B** Expression level of 24 pyroptosis-related DEGs in in GSE142025. **C** mRNA expression of CASP1, CASP8, IL18, and TP53 by qRT-PCR. *P* values were shown as: *P < 0.05; **P < 0.01; ***P < 0.001, n = 3

To further validate the pyroptosis-related genes identified through the above analysis, the human dataset GSE30528 and GSE142025 were checked. Among the 24 DEGs, 4 genes (CASP1, CASP8, IL18, and TP53) were upregulated in GSE30528 (Kidney tissue samples were microdissected glomeruli from DN patients, the same as GSE96804), 10 genes (CASP1, CASP3, CASP4, CASP8, NLRP1, PLCG1, NAIP, ZBP1, IRF1 and TP53) were upregulated and CASP9 was downregulated in GSE142025 (Kidney tissue samples were from advanced DN patients) (Fig. 3A, B). At last, 4 pyroptosis-related genes (CASP1, CASP8, IL18, and TP53) with the same expression level in GSE96804 and GSE30528 were verified using glomerular mesangial cells by qRT-PCR. As illustrated in Fig. 3C, CASP1, CASP8, IL18, and TP53 expression were significantly upregulated in high glucose (HG, 30 mM) group compared with normal glucose (NG, 5.56 mM) group (*P* < 0.05).

### Correlation analysis of pyroptosis-related genes expression in DN

**Fig. 4:**
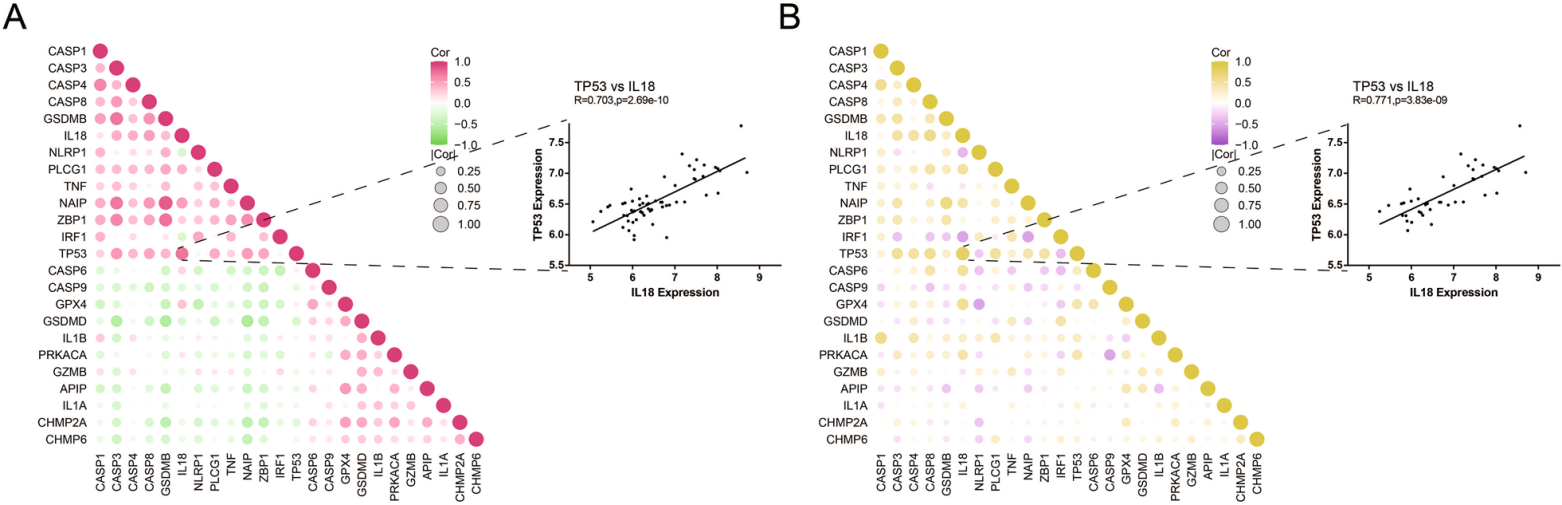
Correlation analysis of pyroptosis-related DEGs in GSE96804. **A** All samples. **B** DN samples. Dot plots of correlation between expression of TP53 and IL18 were displayed on the right side

The rcorr function of “hmisc” package is used to calculate the expression correlation of the 24 pyroptosis-related DEGs in all samples and DN samples of GSE96804. Figure 4 showed that the expression of TP53 and IL18 is significantly positively correlated (all samples: R = 0.703, *P* = 2.69e−10; DN samples: R = 0.771, *P* = 3.83e−09).

### Construction of pyroptosis-related gene predictive model.

**Fig. 5:**
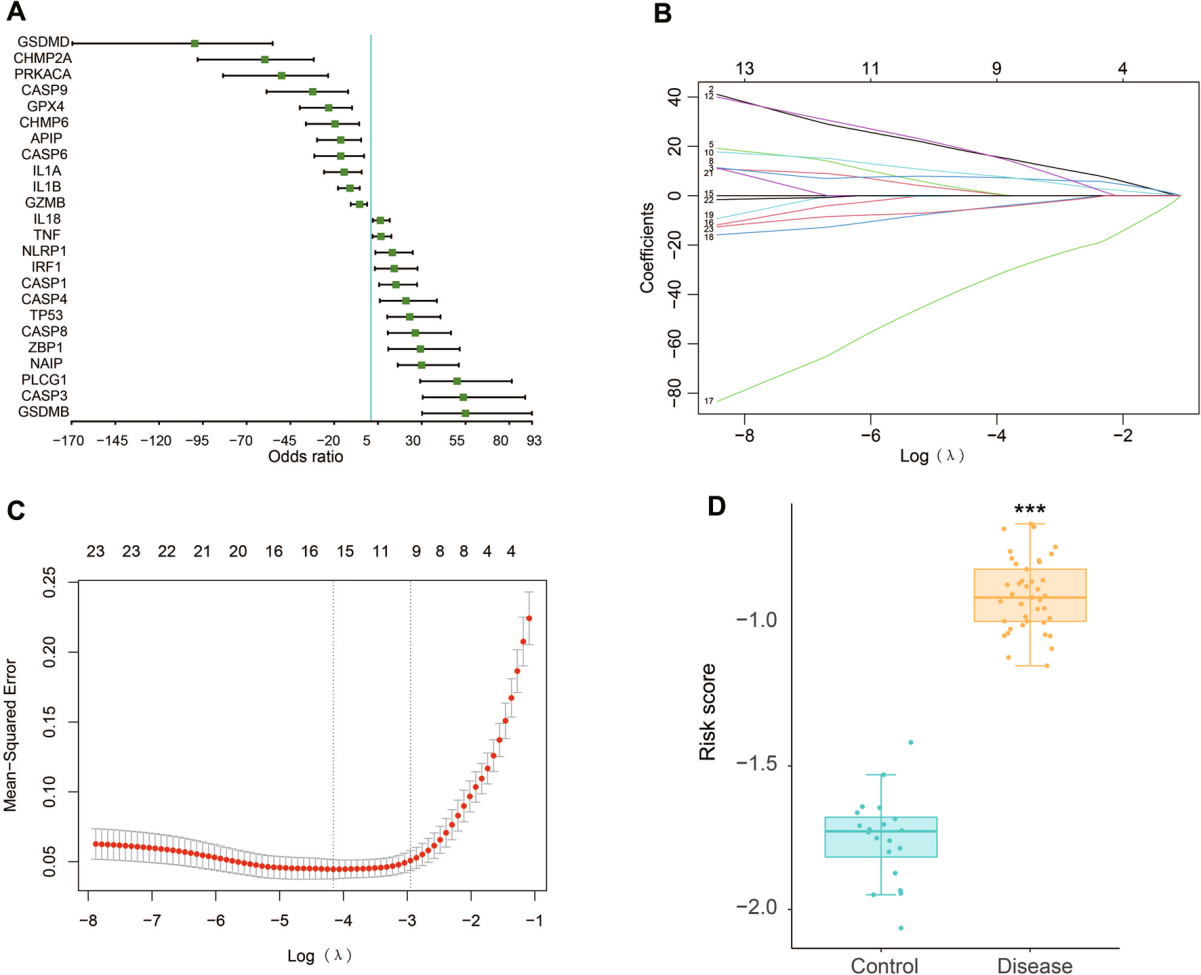
Construction of risk model in the GSE96804 dataset. **A** Univariate logistic regression analysis of the 24 pyroptosis-related DEGs. **B** LASSO regression of the 24 pyroptosis-related DEGs. **C** Cross-validation for tuning the parameter selection in the LASSO regression. **D** Risk score of normal and DN group. *P* values were shown as: ***P < 0.001

Univariate logistic regression analysis was used for screening of DN-related cell pyroptosis-related genes among the above 24 pyroptosis-related DEGs. All 24 genes met the significance of *P* < 0.05 and retained for further analysis (Fig. 5A). Then we applied these genes into LASSO regression algorithm and we incorporated 16 genes into the predictive model according to the optimum λ value (Fig. 5B, C). The risk score was presented as follows: Risk score = (1.33036044153335 * CASP3 exp.) + (0.400394013689692 * CASP4 exp.) + (0.505530396426867 * CASP8 exp.) + (0.0835873650722197 * GSDMB exp.) + (0.265070040429043 * IL18 exp.) + (0.0463927390430767 * NLRP1 exp.) + (0.166092047248583 * PLCG1 exp.) + (0.000505526485423617 * TNF exp.) + (0.650800111035948 * NAIP exp.) + (2.47867874084334 * IRF1 exp.) + (-0.712497977048085 * CASP9 exp.) + (0.470058565874875 * GPX4 exp.) + (-1.6895963489882 * GSDMD exp.) + (-1.03724506279439 * IL1B exp.) + (-1.50927142851797 * PRKACA exp.) + (-0.839345383378387 * CHMP2A exp.). According to the risk score formula, we found that the risk score of the DN group is significantly higher than that of the control group (*P* < 0.001, Fig. 5D).

### Identifying pyroptosis-associated molecular subgroups and differences in the immune microenvironment between subgroups

**Fig. 6:**
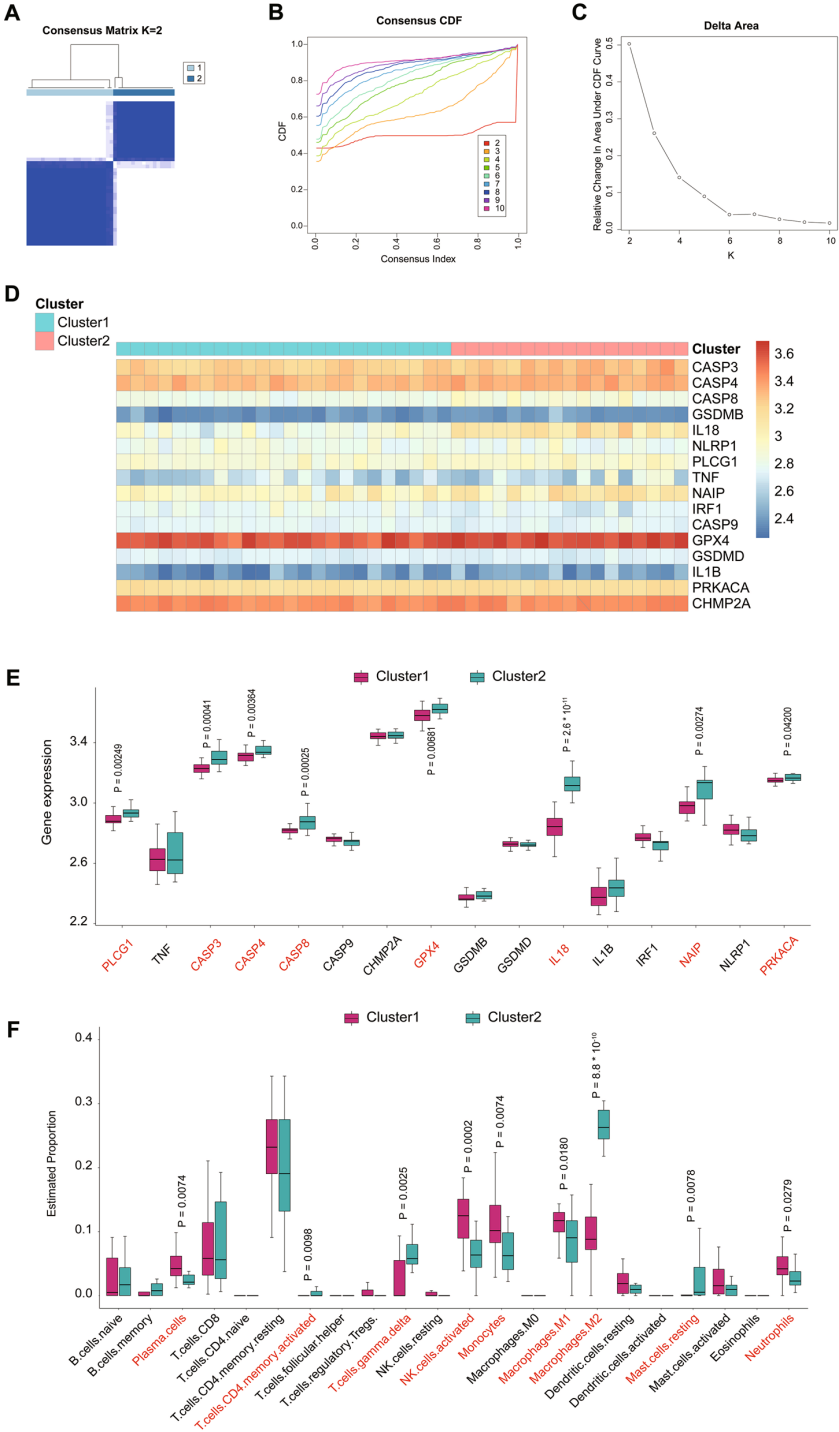
DN classification based on the pyroptosis-related DEGs. **A** 41 DN patients in GSE96804 dataset were grouped into two clusters according to the consensus clustering matrix (k = 2). **B** Consensus among clusters for each category number k. **C** Delta area curves for consensus clustering indicating the relative change in area under the cumulative distribution function (CDF) curve for each category number k compared to k-1. The horizontal axis represents the category number k and the vertical axis represents the relative change in area under CDF curve. **D** Heatmap of the two clusters classified by the 16 pyroptosis-related DEGs. **E** A box plot showed the expression of the 16 pyroptosis-related DEGs. **F** The immune cell infiltration score in two pyroptosis-related clusters

To explore the connections between the expression of the 16 pyroptosis-related DEGs and DN subtypes, we performed a consensus clustering analysis with all 41 DN patients in the GSE96804 dataset. By increasing the clustering variable (k) from 2 to 10, we found that when k = 2, the intragroup correlations were the highest and the intergroup correlations were low, indicating that the 41 DN patients could be well divided into two clusters based on the above16 DEGs (Fig. 6A–C). 24 cases were included in pyroptosis-related Cluster 1 and 17 cases were included in pyroptosis-related Cluster 2. Then we explored the inter cluster expression pattern of the 16 pyroptosis-related genes and found that 8 of the 16 genes (CAPS3, CASP4, CASP8, GPX4, IL18, IRF1, NAIP, PRKACA) have significant expression differences among clusters. Cluster 1 exhibited higher expression levels of IRF1, while Cluster 2 was characterized by enhanced expression of CAPS3, CASP4, CASP8, GPX4, IL18, NAIP and PRKACA (Fig. 6D, E).

Moreover, we used Cell-type Identification By Estimating Relative Subsets Of RNA Transcripts (CIBERSORT) algorithm [26] to calculate the immune infiltration scores of the 41 DN samples between the two clusters in order to determine the relative proportions of 22 infiltrating immune cells. Comparing the scores between Cluster 1 and Cluster 2, it was found that there were significant differences in 9 kinds of cells. Cluster 1 had higher infiltration levels of plasma cells, activated NK cells, monocytes, M1 macrophages and neutrophils, and lower infiltration levels of activated CD4 T cells, gamma delta T cells, M2 macrophages and resting mast cells, while Cluster 2 showed the opposite trends (Fig. 6F).

### Functional analysis of different molecular subtypes

**Fig. 7:**
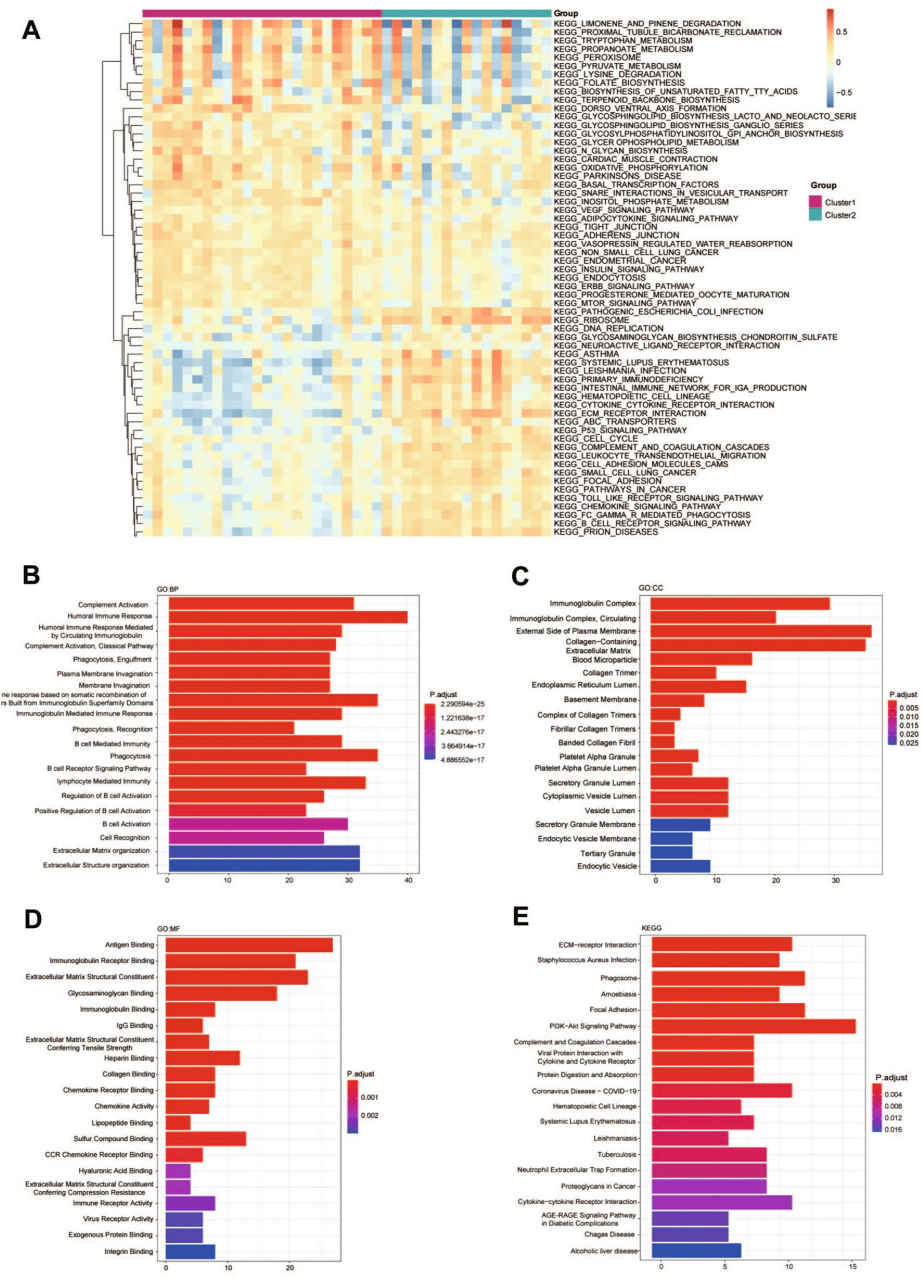
Enrichment analysis in distinct DN clusters. **A** Heatmap of GSVA analysis showed representative Hallmark pathways. **B** GO analysis in biological process (BP). **C** GO analysis in cellular component (CC). **D** GO analysis in molecular function (MF). **E** KEGG pathway enrichment analysis

To explore the differences in biological processes between distinct pattern clusters mediated by pyroptosis, we conducted GSVA enrichment analysis and found that Cluster 1 was primarily enriched in metabolism related pathways (KEGG_TRYPTOPHAN_METABOLISM, KEGG_PROPANOATE_METABOLISM, KEGG_PYRUVATE_METABOLISM), while Cluster 2 was enriched in DNA replication (KEGG_DNA_REPUBLICATION) and p53 signaling pathway (KEGG_P53_SIGNALING_PATHWAY), as shown in Fig. 7A.

Then, we furth identified 250 DEGs of the two clusters using “limma” R package with the screening conditions of *P* < 0.05 and log2(Fold Change) > 1.1. To clarify the biological characteristics of these DGEs, the GO and KEGG pathway analyses were conducted. The significant enrichment results included 231 biological process (BP) terms, 21 cellular component (CC) terms and 38 molecular function (MF) terms. The top 20 significantly enriched terms in BP, MF and CC categories are shown in Fig. 7B–D, which revealed that these DEGs to be enriched in BP including complement activation, humoral immune response, and humoral immune response mediated by circulating immunoglobulin, and CC including immunoglobulin complex, external side of plasma membrane, and collagen-containing extracellular matrix, MF including antigen binding, immunoglobulin receptor binding, and extracellular matrix structural constituent. 25 relevant pathways were obtained by KEGG pathway enrichment. The pathways were enriched into the composition of extracellular structure, activation of immune cells, immunoglobulin mediated immune response, humoral immune response, immunoglobulin complex Cytokine receptor interaction and autoimmune diseases (systemic lupus erythematosus), as shown in Fig. 7E.

### Construction of PPI network based on differential genes and predication of drug-gene interaction

**Fig. 8:**
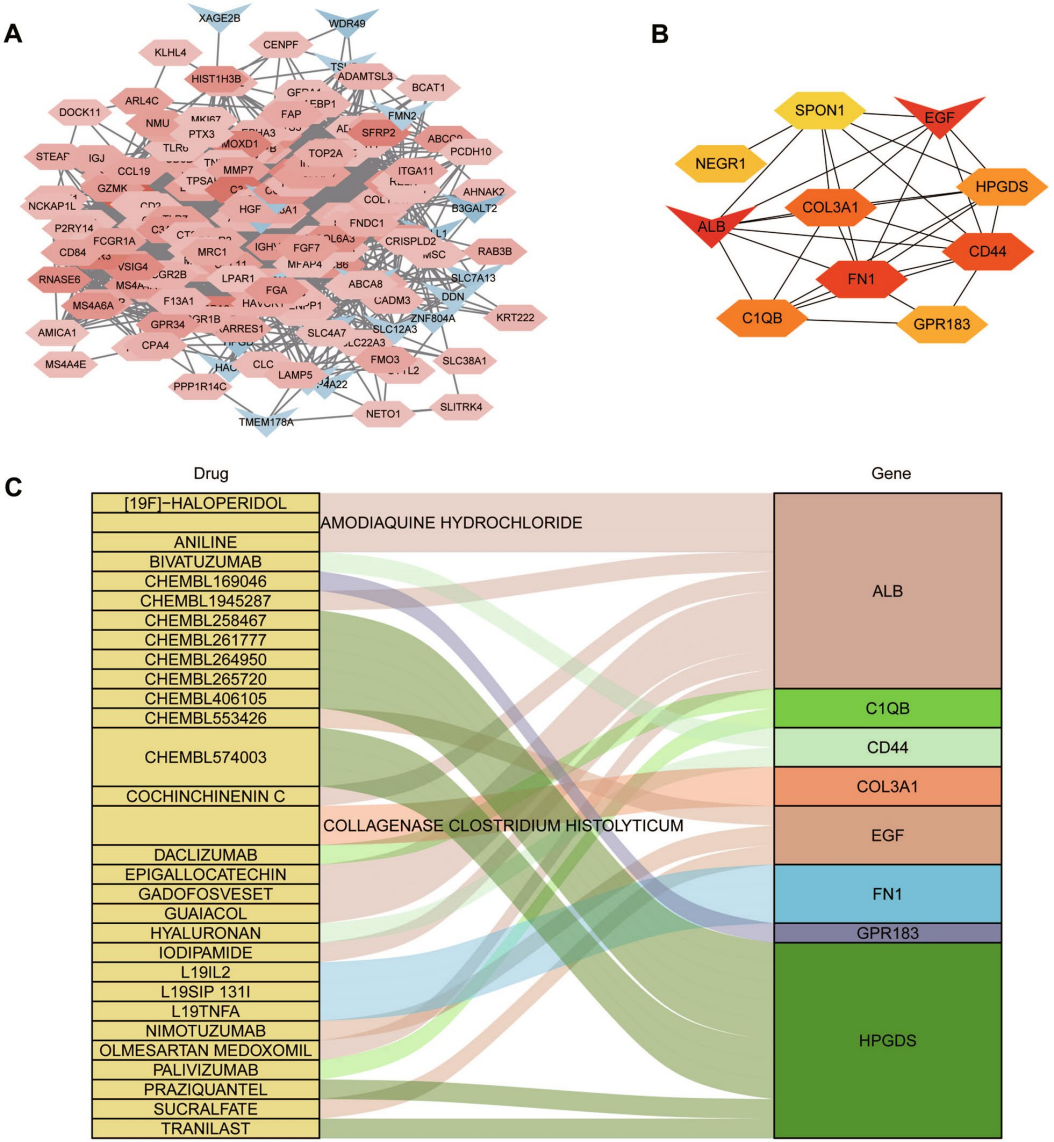
Drug prediction based on hub nodes in differentially expressed gene clusters. **A** PPI network of 250 differentially expressed genes between two distinct clusters. **B** Hub genetic analysis. **C** Drug–gene interaction prediction of hub genes. 10 key genes were targeted in the DGIdb database and top 30 potential target drugs/compounds were predicted from the database

**Fig. 9:**
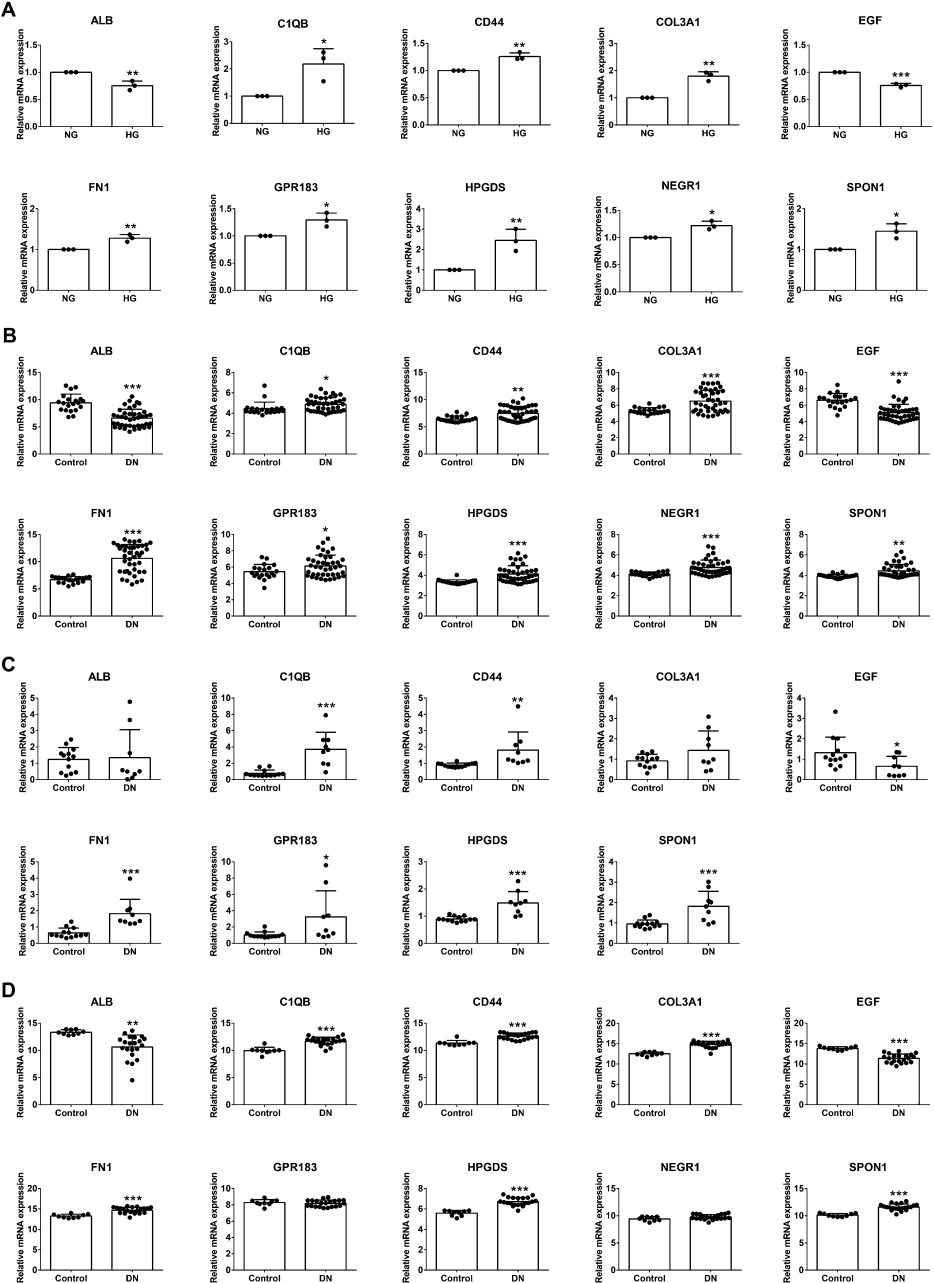
The expression of the identified top 10 hub genes (ALB, C1QB, CD44, COL3A1, EGF, FN1, GPR183, HPGDS, NEGR1 and SPON1). **A** mRNA expression of hub genes from glomerular mesangial cells by qRT-PCR verification. n = 3. **B**–**D** mRNA expression of hub genes in GSE96804 (**B**), GSE30528 (**C**) and GSE142025 (**D**). *P* values were shown as: *P < 0.05; **P < 0.01; ***P < 0.001

To further explore the interactions of these 250 DEGs between the two DN clusters, we conducted a PPI network using Cytoscape software, and 182 nodes plus 3261 edges were obtained (Fig. 8A). Then, the top 10 hub genes therein with the highest score were determined using cytohubba plug-in (Fig. 8B). These hub genes were ALB, C1QB, CD44, COL3A1, EGF, FN1, GPR183, HPGDS, NEGR1 and SPON1, and they were used as the potential druggable targets for DN treatment. The drug–gene interaction results from the DGIdb database revealed 65 potential target drugs/compounds for DN treatment. The top 30 drugs/compounds are displayed in Fig. 8C according to the order of “interaction group score” from the DGIdb database. Of these, 10 drugs targeted ALB, among which had the highest score of prediction; 8 drugs targeted HPGDS, 3 drugs each targeted EGF and FN1, two drugs each targeted C1QB and CD44, and 1 drug each targeted COL3A1 and GPR183. No potential drugs could be identified for NEGR1 and SPON1. At last, the potential targets mentioned above were verified by qRT-PCR. As illustrated in Fig. 9A, C1QB, CD44, COL3A1, FN1, GPR183, HPGDS, NEGR1 and SPON1 expression were significantly upregulated, while ALB and EGF were downregulated in high glucose (HG, 30 mM) group compared with normal glucose (NG, 5.56 mM) group (*P* < 0.05). Moreover, we detected the expression of those genes in samples of DN patients using GSE96804, GSE30528 and GSE142025 datasets (Fig. 9B–D). Most of these data were consistent with our experimental results. Differently, the expression of GPR183 and NEGR1 had no significant difference in GSE142025, the expression of ALB and COL3A1 had no significant difference in GSE30528, and no expression data of NEGR1 was detected in GSE30528.

## Discussion

Pyroptosis, also known as cellular inflammatory necrosis, can release inflammatory factors. Several previous studies have shown inflammation plays a key role in the DN progression and preclinical studies have identified several anti-inflammatory molecules that are effective in decreasing albuminuria and/or proteinuria [9]. Thus, inhibition of pyroptosis-related inflammatory signaling pathways is a promising therapeutic strategy to improve the renal outcome of patients with DN. However, the role of pyroptosis-related genes in DN has not been fully elucidated, and our study aims to shed light on this role.

We first clarified the expression of pyroptosis-related genes in DN. A total of 57 pyroptosis-related genes were obtained and 44 genes expressed in DN and normal kidney tissues using the GSE96804 and GSE30528 datasets. Of these, 13 genes upregulated and 11 genes downregulated in DN compared to normal tissues in training dataset GSE96804. Further validation of the expression level of these 24 DEGs was performed in additional two external datasets (GSE30528 and GSE142025). The results revealed that CASP1, CASP8 and TP53 were upregulated in all 3 datasets. Caspase-1 is a critical molecule for initiating the canonical pathway of pyroptosis. Many studies have shown that Caspase-1/GSDMD pyroptosis pathway was activated in DN [18, 27]. Caspase-8 is an apical caspase participated in a variety of programmed cell death such as apoptosis, necroptosis, etc. [28]. Hsu et al. found that caspase-8 was upregulated in DN podocyte apoptosis triggered by IL-20 [29]. Recent studies revealed significant crosstalk between different forms of cell death [30], and caspase-8 acts as a molecular switch for apoptosis, necroptosis and pyroptosis [31]. These studies suggest caspase-8 mediated pyroptosis might be involved in the pathogenesis of DN. TP53 is a critical tumor suppressor gene that has a multifaceted role in the development of cancer. Zhang et al. suggested TP53 regulated inhibitor of apoptosis 1 (TRIAP1) involved in the development of DN through regulating podocyte apoptosis [32]. However, there is no research focus on the relationship between TP53 and DN. How TP53 changes during pyroptosis remains to be further investigated. Moreover, the expression of TP53 and IL18 is significantly positively correlated in the correlation analysis of pyroptosis-related DEGs (Fig. 3). It has been widely reported that IL-18 upregulation correlates with DN progression [33, 34]. These studies suggest that TP53 mediated pyroptosis may be involved in the development of DN. Finally, the expression of 4 DEGs (CASP1, CASP8, IL18, and TP53) was validated by qRT-PCR and the results were consistent with our integrated analysis (Fig. 3C).

Considering that pyroptosis may be a key regulatory factor for the occurrence and development of DN, we constructed a predictive model using those 24 pyroptosis-related DEGs. Finally, a predictive model with 16 identified pyroptosis genes (CASP3, CASP4, CASP8, GSDMB, IL18, NLRP1, PLCG1, TNF, NAIP, IRF1, CASP9, GPX4, GSDMD, IL1B, PRKACA and CHMP2A) was used to calculate risk score of all samples in GSE96804. Results showed that the risk score of the DN group is significantly higher than that of the control group (*P* < 0.001, Fig. 5D). Furthermore, we classified two distinct DN subtypes based on the expression of the 16 genes listed above. Results shown that most pyroptosis-related regulators exhibited high expression levels in Cluster 2. Currently, increasing studies have proved that pyroptosis is closely related to the activation of immune response [35, 36]. Thus, we further investigated the relationship between pyroptosis clusters and infiltrated immune cells. Compared to Cluster 1, Cluster 2 had higher infiltration levels of activated CD4 T cells, M2 macrophages, gamma delta T cells and resting mast cells, and lower infiltration levels of plasma cells, activated NK cells, monocytes, M1 macrophages and neutrophils, indicating an immunosuppressive microenvironment. GVSA enrichment analysis indicated that Cluster 1 was predominantly enriched in pathways of metabolism and Cluster 2 was enriched in p53 and DNA replication pathways. We further clarified the biological characteristics of 250 DEGs between the two pyroptosis groups. GO and KEGG analyses shown that top DEGs between clusters were enriched in complement activation, humoral immune response and activation of immune cells, suggesting that activation of immune responses may have a potential role in DNs. These results indicated that the enriched pathways and biological functions of pyroptosis clusters are significantly different in DN patients, suggesting that separate treatment strategies should be taken for the different patients.

In addition to the new anti-diabetic drugs that possess markedly cardiovascular and renal protective effects, no novel direct therapies for DN have become commercially available in the past 20 years. In this study, 10 key genes (ALB, C1QB, CD44, COL3A1, EGF, FN1, GPR183, HPGDS, NEGR1 and SPON1) of top DEGs in the two pyroptosis clusters were identified and used for predicting drug-gene interactions. ALB, known as albumin, is the main component of serum protein and associates with maintaining oncotic pressure and systemic inflammatory reaction [37]. Patients with DN usually have clinical symptoms of hypoproteinemia and albuminuria. Moreover, a recent study reported that the level of serum albumin is associated with prognosis in DN patients [38]. C1QB, encodes the B-chain polypeptide of complement C1q and participates in immune response lectin-induced complement pathway and innate immune system [39]. Jiang et al. found that C1QB is highly expressed in DN and emphasize the roles of C1 and local complement activation in DN [40]. CD44, a well-known glycoprotein on cell surface, is involved in many biological processes, such as cell migration, proliferation, lymphocyte activation, etc. [41]. Research has shown that CD44 plays a vital role in the pathogenesis of experimental crescentic glomerulonephritis and collapsing focal segmental glomerulosclerosis [42]. COL3A1, known as collagen III, α1, which is an important stroma component. It has been reported that collagenofibrotic (collagen III) glomerulopathy is closely related to the progression of DN [43]. EGF, epidermal growth factor, the main function of which is to promote DNA synthesis and mitosis after specific binding with EGF receptors on target cells. Recently, EGF was considered as a urine biomarker in DN patients [44]. FN1 encodes fibronectin which is one of the important components of the extracellular matrix (ECM). Studies have revealed that FN1 plays a key role in fibrosis and endothelial cell dysfunction in DN [45, 46]. GPR183, G protein receptor 183 expressed on immunocyte such as T cells, B cells, dendritic cells and macrophages, is mainly involved in intracellular calcium release after ligand activation [47]. HPGDS, hematopoietic prostaglandin D synthaseis, a rate limiting enzyme for the synthesis of prostaglandin D2 (PGD2). Zhang et al. pointed out that PGD2 may play a role in the pathogenesis of chronic kidney diseases while the underlying mechanism was not well elucidated [48]. NEGR1, known as neuronal growth regulator 1, is a cell-adhesion molecule of the immunoglobulin LON (IgLON) family which is closely related to synaptic structure and function [49]. SPON1, encodes the protein spondin 1 which is a member of the thrombospondin family, involves in many biological processes, such as angiogenesis, proteolysis, etc. [50]. However, it is worth noting that there is few research about the roles of GPR183, HPGDS, NEGR1 and SPON1 on DN progression.

Furthermore, we obtained the drug–gene interaction results from the DGIdb database. A total of 65 potential drugs or compounds for DN treatment were presented. Most of them target the ALB gene. We examined these 65 candidates from ClinicalTrials.gov (Accessed on 11 April 2023), which is the largest clinical trials database containing over 448 116 trials worldwide. Five targetable drugs (Epigallocatechin, Olmesartan medoxomil, Silver and Naproxen targeting ALB; Glutathione targeting HPGDS) were found to be used for DN treatment. While there is no drugs could be predicted for the NEGR1 and SPON1 genes. NEGR1has been reported to regulate LPS-induced endothelial-mesenchymal transition [51] and involve the progression and metastasis of kidney cancer [52]. One of the proteins encoded by SPON1 is spondin1, a secreted ECM protein, which has been reported to be abnormally expressed in numerous human diseases [53, 54]. One of the major pathological changes in DN is excessive deposition of ECM [55] and much research has suggested that targeting the ECM would facilitate the development of novel therapeutic strategies for DN [56–58]. Thus, these two candidate genes may be potential targets for DN treatment, which is need to be evaluated in further studies.

Our study has some limitations. First, our study lacked relevant information on the patients' clinical characteristics, such as age, gender, and disease duration, which could potentially affect gene expression. Additionally, larger numbers of DN patient samples need to be taken into account to validate the stability of the research results. Moreover, future studies should include in vitro and in vivo experiments to validate the therapeutic potential of the identified compounds.

In summary, we performed a comprehensive and systematic bioinformatics analysis and identified key genes related to pyroptosis in DN patients. Our results also identified 65 potential therapeutical agents/compounds for DN according to the key genes between two pyroptosis related DN subtypes. Further study should be conducted to verify the effectiveness of the agents/compounds for DN.

## Supplementary Information

**Additional file 1: Table S1.** Pyroptosis-related genes from literature and databases. **Figure S1. **Pyroptosis-related genes obtained from literature, Reactome gene sets and GOBP gene sets from MSigDB database. (A) Venn (B) Column.

## Data Availability

The RNA expression data could be download at https://www.ncbi.nlm.nih.gov/geo/ and all data generated during this study are included in this article and its Additional files. All data are available from the corresponding author on reasonable request.

## Abbreviations

DN: Diabetic nephropathy
DM: Diabetes mellitus
LASSO: Least absolute shrinkage and selection opera
DEGs: Differentially expressed genes
PPI: Protein–protein interaction
DKD: Diabetic kidney disease
EGF: Epidermal growth factor
ESRD: End stage renal disease
GSDMD: Gasdermin
DGIdb: Drug Gene Interaction Database
AUC: Area under the ROC curve
CIBERSORT: Estimating Relative Subsets Of RNA Transcripts
BP: Biological process
CC: Cellular component
MF: Molecular function
TRIAP1: TP53 regulated inhibitor of apoptosis 1
ROC: Receiver operating characteristic
ECM: Extracellular matrix
MSigDB: Molecular Signatures Database
CDF: Cumulative distribution function
GSVA: Gene set variation analysis
GO: Gene Ontology
KEGG: Kyoto Encyclopedia of Genes and Genomes
PGD2: Prostaglandin D2
STRING: Search Tool for the Retrieval of Interacting Genes
