# Supplementary material for: Identification of pyroptosis-related genes and potential drugs in diabetic nephropathy
Source: J Transl Med. 2023 Jul 21;21:490. doi: 10.1186/s12967-023-04350-w (PMC10360355; doi:10.1186/s12967-023-04350-w)
Supplement: Supplementary file 1 — Additional file 1: Table S1. Pyroptosis-related genes from literature and databases. Figure S1. Pyroptosis-related genes obtained from literature, Reactome gene sets and GOBP gene sets from MSigDB database. (A) Venn (B) Column. [file 12967_2023_4350_MOESM1_ESM.docx]

Table S1 Pyroptosis-related genes from literature and databases.

| Gene | Full-names | Source |
| --- | --- | --- |
| BAK1 | BCL2 antagonist/killer 1 | GOBP |
| BAX | BCL2 associated X | GOBP |
| CHMP2A | Charged multivesicular body protein 2A | GOBP |
| CHMP2B | Charged multivesicular body protein 2B | GOBP |
| CHMP3 | Charged multivesicular body protein 3 | GOBP |
| CHMP4A | Charged multivesicular body protein 4A | GOBP |
| CHMP4B | Charged multivesicular body protein 4B | GOBP |
| CHMP4C | Charged multivesicular body protein 4C | GOBP |
| CHMP6 | Charged multivesicular body protein 6 | GOBP |
| CHMP7 | Charged multivesicular body protein 7 | GOBP |
| CYCS | Cytochrome c, somatic | GOBP |
| HMGB1 | High mobility group box 1 | GOBP |
| IL1A | Interleukin 1 alpha | GOBP |
| IRF1 | Interferon regulatory factor 1 | GOBP |
| IRF2 | Interferon regulatory factor 2 | GOBP |
| TP53 | Tumor protein p53 | GOBP |
| TP63 | Tumor protein p63 | GOBP |
| CASP6 | Cysteine-aspartic acid protease 6 | literature |
| CASP9 | Cysteine-aspartic acid protease 9 | literature |
| GPX4 | Glutathione peroxidase 4 | literature |
| IL6 | Interleukin 6 | literature |
| NLRP2 | NLR family pyrin domain containing 2 | literature |
| NLRP3 | NLR family pyrin domain containing 3 | literature |
| NLRP6 | NLR family pyrin domain containing 6 | literature |
| NLRP7 | NLR family pyrin domain containing 7 | literature |
| NOD1 | Nucleotide binding oligomerization domain containing 1 | literature |
| NOD2 | Nucleotide binding oligomerization domain containing 2 | literature |
| PJVK | Pejvakin | literature |
| PLCG1 | Phospholipase C gamma 1 | literature |
| PRKACA | Protein kinase cAMP-activated catalytic subunit alpha | literature |
| PYCARD | PYD and CARD domain containing | literature |
| SCAF11 | SR-related CTD associated factor 11 | literature |
| TIRAP | TIR domain containing adaptor protein | literature |
| TNF | Tumor necrosis factor | literature |
| APIP | APAF1 interacting protein | REACTOME |
| DHX9 | DExH-box helicase 9 | REACTOME |
| GZMA | Granzyme A | REACTOME |
| NAIP | NLR family apoptosis inhibitory protein | REACTOME |
| NLRP9 | NLR family pyrin domain containing 9 | REACTOME |
| ZBP1 | Z-DNA binding protein 1 | REACTOME |
| CASP3 | Cysteine-aspartic acid protease 3 | GOBP, literature |
| CASP5 | Cysteine-aspartic acid protease 5 | GOBP, literature |
| ELANE | Elastase, neutrophil expressed | GOBP, literature |
| IL18 | Interleukin 18 | GOBP, literature |
| IL1B | Interleukin 1 beta | GOBP, literature |
| GZMB | Granzyme B | GOBP, REACTOME |
| AIM2 | Absent in melanoma 2 | literature, REACTOME |
| CASP8 | Cysteine-aspartic acid protease 8 | literature, REACTOME |
| GSDMA | Gasdermin A | literature, REACTOME |
| GSDMB | Gasdermin B | literature, REACTOME |
| GSDMC | Gasdermin C | literature, REACTOME |
| NLRC4 | NLR family CARD domain containing 4 | literature, REACTOME |
| NLRP1 | NLR family pyrin domain containing 1 | literature, REACTOME |
| CASP1 | Cysteine-aspartic acid protease 1 | GOBP, literature, REACTOME |
| CASP4 | Cysteine-aspartic acid protease 4 | GOBP, literature, REACTOME |
| GSDMD | Gasdermin D | GOBP, literature, REACTOME |
| GSDME | Gasdermin E | GOBP, literature, REACTOME |


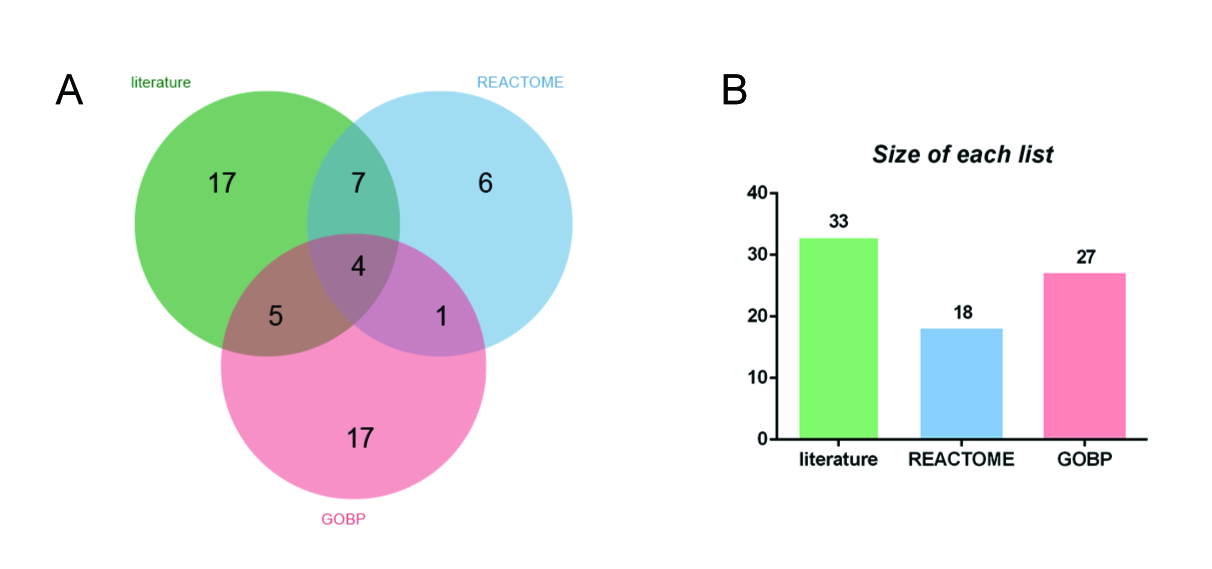


**Figure S1** Pyroptosis-related genes obtained from literature, Reactome gene sets and GOBP gene sets from MSigDB database. (A) Venn (B) Column.
